# Nonlinear Guided-Wave Mixing for Condition Monitoring of Bolted Joints

**DOI:** 10.3390/s21155093

**Published:** 2021-07-27

**Authors:** Juan Carlos Pineda Allen, Ching Tai Ng

**Affiliations:** School of Civil, Environmental & Mining Engineering, The University of Adelaide, Adelaide, SA 5005, Australia; juancarlos.pinedaallen@adelaide.edu.au

**Keywords:** wave mixing, bolt loosening, bolted joint, Lamb wave, combination harmonic, guided wave, nonlinear

## Abstract

Bolted joints are fundamental to numerous structural components in engineering practice. Nevertheless, their failure or even their loosening can lead to insufficient performance and reduced structural safety. This study presents a theoretical development and experimental investigation into nonlinear guided-wave mixing for integrity monitoring of bolted joints in plates. Combinational harmonics generated due to nonlinear Lamb wave mixing and contact acoustic nonlinearity at the bolted joints were used to evaluate the applied torque level in the joint. The area of the power spectral density in the region of the sum combinational harmonic bandwidth is found to be highly correlated to the applied torque level at the joint. Moreover, the effect of the number of cycles and thus the time duration of the excitation is investigated. The results show that the combinational harmonics remain robust for different numbers of cycles in detecting bolt loosening. The findings presented in this study also provide physical insight into the phenomena of nonlinear Lamb wave mixing for evaluating applied torque in bolted joints, and the results help further advance the use of nonlinear guided waves for damage detection.

## 1. Introduction

To evaluate changes in structural performance, the capabilities of both structural health monitoring (SHM) and non-destructive testing (NDT) have been joined to be developed and implemented in real structures. Integrity and safety are paramount to any structural components; hence, there is great interest in early damage and degradation detection. Bolted structural connection is an efficient and versatile connection technique widely used in various engineering structures such as bridges [1], wind turbines [2], and buildings [3]. In these types of structures, the unsatisfactory performance of connections can drastically compromise the structure. Previous studies revealed that bolt loosening can significantly decrease the fatigue life of bolted joints [4].

A direct approach to monitor bolted joints is through the installation of load cells or strain gauges at the bolts. However, this approach requires as many load cells as bolts to monitor the bolted connections. This significantly increases inspection and operational cost for structures. Alternatively, indirect methods have also been studied in the literature. Traditional ultrasonic techniques have been investigated for axial force monitoring in bolts [5,6]. Transmittance [7], impedance [8], and coda wave [9] methods have also been investigated.

### 1.1. Guided Waves for Bolt Condition Assessment

Contrary to traditional NDT techniques, methods based on guided waves (GWs) have the ability to inspect large and inaccessible areas. They have attracted significant research interest for NDE and SHM in recent years. GWs have the ability to propagate in different types of structural elements, such as beams [10], bars [11], pipes [12], and plates [13], and their multimodality [14,15] provides flexibility in inspecting structures. Wang et al. [16] demonstrated that the energy propagated across the bolt can potentially indicate the bolt’s status. They also studied a time-reversal linear GW-based method and showed that the bolt preload values correlate with the peak amplitude for the focused signal [17]. However, nonlinear methods possess advantages over their linear counterparts. Linear features, e.g., time-of-flight, of the scattered waves or mode-converted waves from damage are difficult to extract when there are many wave reflections in the time-domain signals. At the very early stage, time-domain features for linear GW techniques may not be sufficient to detect bolt-loosening effects.

### 1.2. Nonlinear Features of Guided Waves

It was demonstrated that nonlinear features can potentially outperform linear techniques for early bolt-loosening detection. Damage indexes were developed for both linear and nonlinear acoustic/ultrasound approaches, and those were fit by hyperbolic tangent functions [18]. An impact-modulation technique showed a correlation between a modulation index and the bolt condition. It was demonstrated that this index is sensitive to the wave-actuating and -sensing location [19]. Based on the overall dynamic behaviour of the structure, a linear approach and nonlinear vibro-acoustic modulation technique study was conducted in [20]. Van de Abeele et al. [21] illustrated the use of nonlinear wave modulation and explored its benefits in detecting crack damage in different materials. Further wave modulation studies with concrete [22] and composite laminates [23] focusing on sideband peak count were also conducted. The aforementioned techniques rely on sideband generation. However, shakers [20] or impact hammers [19] are essential devices for the impact-modulation and vibro-acoustic modulation techniques. They significantly increase the cost of these techniques and restrict the applicability of these techniques for in situ monitoring.

Analogous to bulk waves, nonlinear GW-based methods have proven more effective compared to linear GW-based methods [24,25], with the benefits of GW methods mentioned earlier, such as increased propagation distances and accessibility. More recently, Lissenden et al. [26] systematically presented the use of nonlinear GWs for NDT, paying special attention to early material degradation detection. They rely on nonlinear acoustic phenomena and are sensitive in detecting early-stage fatigue [27,28], local debonding [29], and delamination [30]. In the literature, there were very few studies that investigated the use of nonlinear GWs for bolt joints monitoring. Yang et al. [31] investigated second harmonic generation due to fatigue crack. They showed that it is possible to differentiate cases when the bolted joint is weakened by fatigue crack. However, the magnitude of the second harmonic is usually very small, which makes it hard to accurately measure. The testing equipment can also introduce nonlinearities that mask harmonics generated by damage-related nonlinearities. Contact between the specimens and the probing transducers can similarly create non-damage-related nonlinearities.

### 1.3. Guided-Wave Mixing

Given the multimodal nature of guided waves, and the existence of higher-order propagation modes, limited work in the field of nonlinear guided-wave mixing can be found in the literature, aiming at fundamentally understanding the mixing phenomenon as in [32,33,34,35] and some types of damage-related mechanisms [36,37,38]. Croxford et al. [39] investigated material degradation detection using wave mixing, and Jingpin et al. investigated the use of wave mixing to detect fatigue crack [40] and thermal corrosion [41] damage in steel specimens. These studies demonstrated the advantages of wave-mixing techniques in detecting micro-cracks, fatigue, and plasticity damage. Even though researchers have explored the use of bulk waves in wave mixing, conventional ultrasonics can inspect only the area covered by the transducer. This is not cost-effective, and defects or damage can sometimes be missed.

Further research is required not only to understand the mixing phenomenon but also to appreciate and benefit from the advantages of nonlinear wave mixing. The need for premature bolt-loosening detection in structures by an inexpensive, reliable, and prompt detection method has motivated this research. Nonlinear guided-wave mixing in bolted structural joints has not been fully investigated in the literature. As such, this study experimentally explores Lamb wave mixing on a steel bolted joint to demonstrate that bolt-loosening effects can be correlated to the combined sum harmonic. Moreover, the use of small and low-cost piezoceramic transducers, instead of traditional ultrasonic transducers, broadens the applicability of the proposed approach to allow integration into in situ NDT and SHM systems. Using nonlinear guided-wave-mixing techniques, the advantages of mid- to long-range inspection would reduce inspection times, resulting in lower related costs, with the added ability to detect early-stage damage [42,43]. This study aims to investigate the use of mixed-frequency responses for monitoring bolted joints. The study focuses on using combined frequency responses for assessing the condition of the bolted joint.

The paper is organised as follows. The first section provides a theoretical framework for the wave-mixing phenomenon. The following section outlines the experimental setup, in which the specimen is described and the wave-actuation and -sensing approach is presented. The excitation signal selection is described in the next section, following the mechanisms for the applied torque studies. This is followed by the results comparing the signals in the time domain with their limitations and the analysis of the proposed mixed-frequency technique. The effect of the number of cycles is studied next, and this study is then finished with concluding remarks.

## 2. Theoretical Background

When an incident GW containing two sinusoidal pulses propagates through a pristine material, the frequency spectrum of the received wave ideally contains frequency components corresponding only to the central frequencies of the two incident sinusoidal signals. However, when the wave travels through a region where a source of nonlinearity is present (e.g., damage- or material-related), higher and combinational harmonics are present in the amplitude spectra of the received wave pulses, in addition to components at the central frequencies of the two incident sinusoidal pulses. The explanation for this phenomenon is that the incident sinusoidal pulses interact with the nonlinearity source and generate the higher and combinational harmonics.

The bolted joint investigated in this paper is considered as a contact interface whose pressure between contact faces varies according to the applied force between the bolt and nut. A simplified approach is used in this study. We consider the joint behaving as a single-degree-of-freedom system with bilinear stiffness [19,44] subjected to an excitation consisting of two sinusoidal forces, each one corresponding to each sinusoidal wave. Consider the following input excitation that consists of two tone-burst pulses:

(1) $$P\left(t\right)=P_{a}\cos\omega_{a}t\left(1-\frac{\omega_{a}t}{N_{a}}\right)+P_{b}\cos\omega_{b}t\left(1-\frac{\omega_{b}t}{N_{b}}\right)$$

where *P_a_* and *P_b_* are the individual forces (with their corresponding amplitudes). *ω_a_* and *ω_b_* are the central frequencies, and *N_a_* and *N_b_* are the corresponding numbers of cycles. *t* represents the time. The equation of motion of the system can be written as follows [20]:

(2) $$P_{a}\cos\omega_{a}t\left(1-\cos\frac{\omega_{a}t}{N_{a}}\right)+P_{b}\cos\omega_{b}t\left(1-\cos\frac{\omega_{b}t}{N_{b}}\right)=m\ddot{y}+k_{1}y-\varphi k_{2}y^{2}$$

where *m* denotes the mass and *φ* is used to scale the nonlinear part in the perturbation solution. 

The linear and nonlinear contact stiffnesses are represented by *k*_1_ and *k*_2_, respectively. The incident wave would interact with the imperfect contact interface, and the solution to Equation (2) using perturbation theory can be expressed as:

(3) $$y=y_{l}+y_{sh}+y_{ch}$$

which consists of the linear response *y_l_* and nonlinear responses from second and combinational harmonics, *y_sh_* and *y_ch_*, respectively. Substituting Equation (3) into Equation (2), we obtain the following relation:

(4) $$m{\ddot{y}}_{l}+k_{1}y_{l}=P_{a}\cos\omega_{a}t\left(1-\cos\frac{\omega_{a}t}{N_{a}}\right)+P_{b}\cos\omega_{b}t\left(1-\cos\frac{\omega_{b}t}{N_{b}}\right)$$

(5) $$m{\ddot{y}}_{sh}+k_{1}y_{sh}-k_{2}y_{l}^{2}=0$$

(6) $$y=y_{l}+y_{sh}+y_{ch}$$

**Figure 1:**
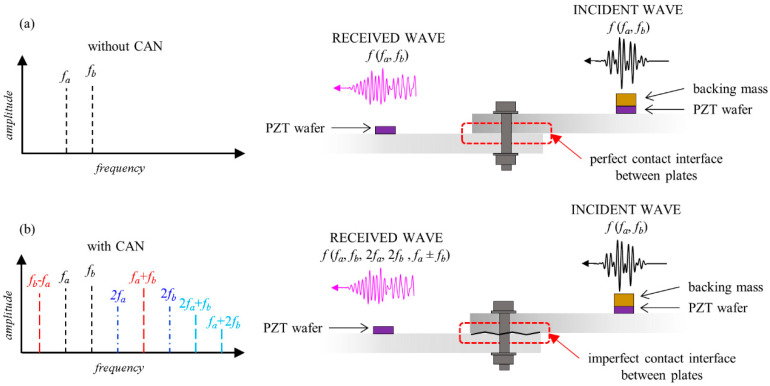
Contact acoustic nonlinearity phenomenon in the bolted joint with (**a**) perfect contact interface and (**b**) imperfect contact interface.

Ignoring the transient components, and making the coefficients equal, linear and nonlinear responses are obtained as below:

(7) $$y_{l}=A_{1}\cos\omega_{a}t\left(1-\cos\frac{\omega_{a}t}{N_{a}}\right)+A_{2}\cos\omega_{b}t\left(1-\cos\frac{\omega_{b}t}{N_{b}}\right)$$

(8) $$y_{sh}=B_{1}k_{2}\cos2\omega_{a}t\left(1-\cos\frac{2\omega_{a}t}{N_{a}}\right)+B_{2}k_{2}\cos2\omega_{b}t\left(1-\cos\frac{2\omega_{b}t}{N_{b}}\right)$$

(9) $$y_{ch}=C_{1}k_{2}\cos\left(\omega_{a}+\omega_{b}\right)t\left(1-\cos\frac{\omega_{a+b}t}{N_{a}}\right)+C_{2}k_{2}\cos\left(\omega_{a}-\omega_{b}\right)t\left(1-\cos\frac{\omega_{a-b}t}{N_{b}}\right)$$

where:

(10) $$A_{1}=\frac{P_{a}\left(1-\cos\frac{\omega_{a}t}{N_{a}}\right)}{k_{1}\left(1-\cos\frac{\omega_{a}t}{N_{a}}\right)+m\omega_{a}^{2}\left(\cos\frac{\omega_{a}t}{N_{a}}-1\right)+\frac{m\omega_{a}^{2}}{N_{a}^{2}}\cos\frac{\omega_{a}t}{N_{a}}}$$

(11) $$A_{2}=\frac{P_{b}\left(1-\cos\frac{\omega_{b}t}{N_{b}}\right)}{k_{1}\left(1-\cos\frac{\omega_{b}t}{N_{b}}\right)+m\omega_{b}^{2}\left(\cos\frac{\omega_{b}t}{N_{b}}-1\right)+\frac{m\omega_{b}^{2}}{N_{b}^{2}}\cos\frac{\omega_{b}t}{N_{b}}}$$

(12) $$B_{1}=\frac{0.5A_{1}^{2}{\left(1-\cos\frac{\omega_{a}t}{N_{a}}\right)}^{2}}{k_{1}\left(1-\cos\frac{2\omega_{a}t}{N_{a}}\right)+4m\omega_{a}^{2}\left(\cos\frac{2\omega_{a}t}{N_{a}}-1\right)+\frac{4m\omega_{a}^{2}}{N_{a}^{2}}\cos\frac{2\omega_{a}t}{N_{a}}}$$

(13) $$B_{2}=\frac{0.5A_{2}^{2}{\left(1-\cos\frac{\omega_{b}t}{N_{b}}\right)}^{2}}{k_{1}\left(1-\cos\frac{2\omega_{b}t}{N_{b}}\right)+4m\omega_{b}^{2}\left(\cos\frac{2\omega_{b}t}{N_{b}}-1\right)+\frac{4m\omega_{b}^{2}}{N_{b}^{2}}\cos\frac{2\omega_{b}t}{N_{b}}}$$

(14) $$C_{1}=\frac{A_{1}A_{2}\left(1-\cos\frac{\omega_{a}t}{N_{a}}\right)\left(1-\cos\frac{\omega_{b}t}{N_{b}}\right)}{k_{1}\left(1-\cos\frac{\omega_{a+b}t}{N_{a}}\right)+m\omega_{a+b}^{2}\left(\cos\frac{\omega_{a+b}t}{N_{a}}-1\right)+\frac{m\omega_{a+b}^{2}}{N_{a}^{2}}\cos\frac{\omega_{a+b}t}{N_{a}}}$$

(15) $$C_{2}=\frac{A_{1}A_{2}\left(1-\cos\frac{\omega_{a}t}{N_{a}}\right)\left(1-\cos\frac{\omega_{b}t}{N_{b}}\right)}{k_{1}\left(1-\cos\frac{\omega_{a-b}t}{N_{b}}\right)+m\omega_{a-b}^{2}\left(\cos\frac{\omega_{a-b}t}{N_{b}}-1\right)+\frac{m\omega_{a-b}^{2}}{N_{b}^{2}}\cos\frac{\omega_{a+b}t}{N_{b}}}$$

where *ω_(a±b)_* = *ω_a_±ω_b_*. It can be seen from Equation (9) that the magnitude of the combinational harmonics is proportional to the nonlinear stiffness *k*_2_ and thus related to the applied torque at the bolted joint. The contact mechanism between interfaces during motion generates the contact acoustic nonlinearity (CAN). The amplitude spectra of the response contain three components. They are: (i) the linear component as shown in Equation (7) that is related to the input frequencies; (ii) a nonlinear component as shown in Equation (8) that consists of higher harmonics; and (iii) a nonlinear component as shown in Equation (9) that consists of combinational harmonics resulting from guided-wave mixing. This phenomenon is schematically illustrated in Figure 1. For the incident wave travelling through a linear medium, the response spectrum of the received wave would contain frequency components corresponding only to the two incident waves as shown in Figure 1a. In contrast, in the presence of a nonlinearity source, such as the imperfect contact interface between plates, the response spectrum of the received wave would contain higher-order harmonics such as second harmonics and combined harmonics, as shown in Figure 1b.

## 3. Experimental Setup

### 3.1. Specimen Description

**Figure 2:**
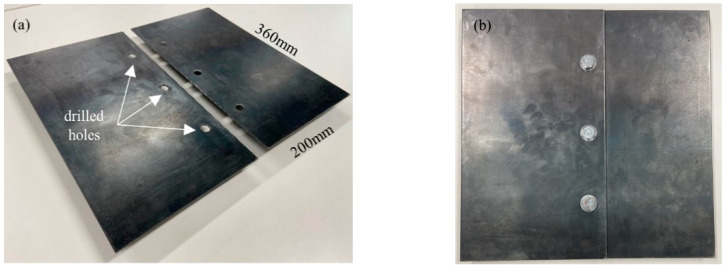
Steel specimens (**a**) before joint; (**b**) after bolted joint.

Steel plates were chosen in this study, which are among the most commonly used materials in the civil and mechanical engineering industry. In general, the findings of this study are applicable to other metallic specimens. The experiments for demonstrating the proposed method were conducted on bolted joints composed of two steel plates, with each of them having in-plane dimensions of 200 mm × 360 mm and thickness of 3 mm, as shown in Figure 2. Both plates are made of G250 mild steel, whose material properties are Young Modulus *E*_s_ = 205 GPa; density *ρ*_s_ = 7820 kg/m^3^; and Poisson ratio *ν*_s_ = 0.29. Each plate has three 10 mm drilled holes, and M10 bolts and nuts were used to join the plates with 40 mm overlap. 

A digital torque wrench, Sidchrome SCMT26952, was used to gradually tighten the bolts. The torque wrench sensitivity was ± 2%. Different torque levels, eleven in total, were applied, and signals were measured at each torque. Minimum applied torque was 20 Nm and maximum was 70 Nm, in 5 Nm steps. Once the eleven levels of applied torque were applied, the bolts were loosened, and the process was repeated for the same eleven levels of the applied torque. The experiment was repeated five times independently. The first part of our analysis is conducted for a single measurement to provide an illustration of the signal quality and the proposed method, and the study finally presents the data for the five independent measures.

### 3.2. Equipment Setup

**Figure 3:**
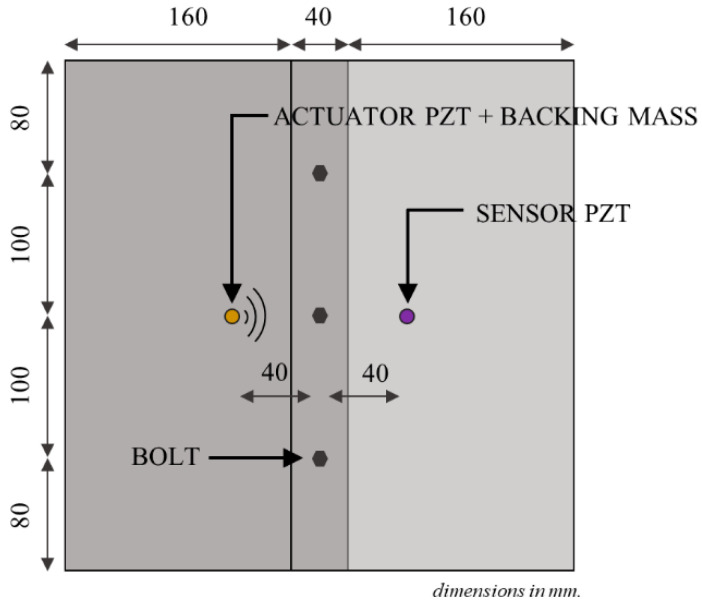
Schematic diagram of the actuating and sensing arrangement.

A circular piezoceramic transducer (PZT) with 5 mm diameter and 2 mm thick was bonded to one of the steel plates using silver conductive epoxy at a distance of 40 mm from the centre of the machined bolt hole. A pitch-catch GW excitation and sensing approach is used in this study. To increase the out-of-plane excitability of the actuator, a brass backing mass was bonded to the top of the transducer using the same conductive epoxy. Another piezoceramic transducer (5 mm diameter and 2 mm thick) was bonded to the other steel plate to receive the actuated wave signal. Using the pitch-catch approach, the signal received by the sensor was expected to carry information from the bolted joint as this signal was generated at the left-hand side of the plate, passed through the bolted joint, and was measured by the sensor located at the right-hand side. A schematic diagram of the actuating and sensing arrangement is shown in Figure 3.

**Figure 4:**
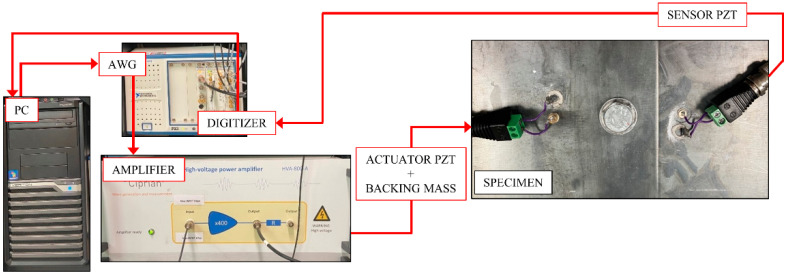
Signal generation and acquisition setup.

An NI PXI-5412 arbitrary wave generator (AWG) was used to generate the excitation signal, which was then fed to a high-power signal amplifier. The signal consists of two sinusoidal tone-burst pulses modulated by Hann windows. These two pulses merged into one signal before sending it to the amplifier. The signal was amplified up to 120 V using a CIPRIAN HVA-400 amplifier and then sent to the actuator. The acquisition was averaged 500 times to improve the signal-to-noise ratio of the measured wave signal. The sensor was connected to an NI PXI-5122 digitiser, and the digitised data were sent to the computer for post-processing. The experimental setup is shown in Figure 4.

### 3.3. Excitation Signal and Frequency Selection for Wave Mixing

Two sinusoidal tone-burst pulses with different central frequencies were merged into one single excitation signal before sending to the amplifier. Preliminary tests with single-frequency pulses were first conducted to evaluate the single-frequency response of the piezoceramic transducers. After that, different frequency combinations were examined to find a suitable frequency combination for the tests. Central frequencies of both pulses were chosen so that (i) the sum combinational frequency would not be a multiple of any of the input frequencies and, (ii) the frequency response of the piezoceramic transducers would be optimised. The combinational harmonic investigated in this study is generated by contact nonlinearity at the bolted joint due to bolt loosening. This is not required to fulfil the internal resonance conditions necessary for evaluating material nonlinearity. One of the sinusoidal pulses was at 110 kHz with 10 cycles, whereas the other sinusoidal pulse was at 160 kHz with 14 cycles. Then, both sinusoidal pulses were added together. The number of cycles was selected so that the durations of both single-frequency sinusoidal tone-burst pulses would be the same and have similar energy content.

## 4. Results

**Figure 5:**
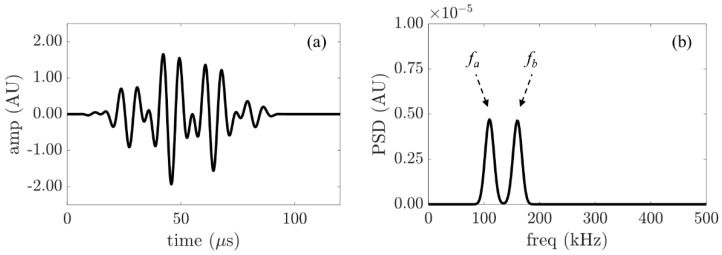
(**a**) Typical time history of the input signal created by merging two signals at different frequencies; (**b**) Typical spectrum of the input signal.

**Figure 6:**
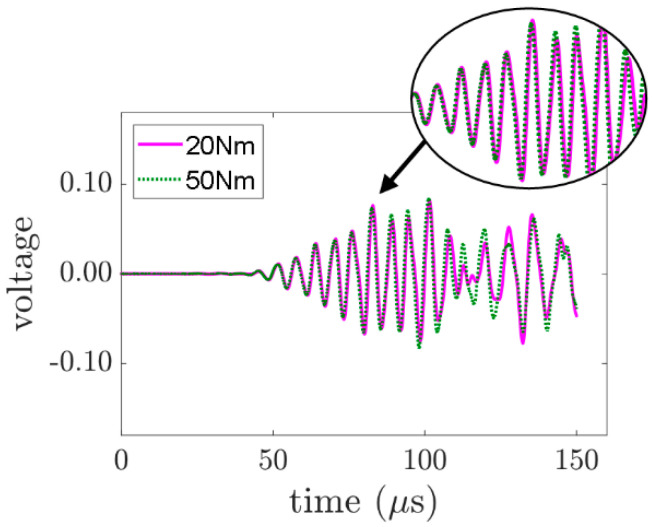
Measured responses of two levels of applied torque.

A typical input signal for wave mixing is shown in Figure 5, which combines two sinusoidal tone bursts with excitation frequencies as described in the last section. The corresponding received signal, which passed through the bolted joint, is shown in Figure 6. The figure shows the signals of two levels of applied torque.

Signals in the time domain can also be compared to some extent. In Figure 6, the received signal for an applied torque of 20 Nm and 50 Nm are plotted together. It can be observed that the signal that travelled through the bolted joint with a greater magnitude of applied torque arrives slightly faster than that which travelled through the bolted joint with a lesser magnitude of applied torque. This can be explained by the fact that when the applied torque of the bolts is increased, the bolted joints tighten the plates. They are in a full-contact situation, and the plates behave like an integrated solid element. Hence, the wave propagates faster. On the other hand, when the bolts are loosened, the interfacial contact between the plates is reduced, and the plates jointed by bolt are less similar to a single solid element. Hence, the wave takes a longer time to arrive at the sensor.

**Figure 7:**
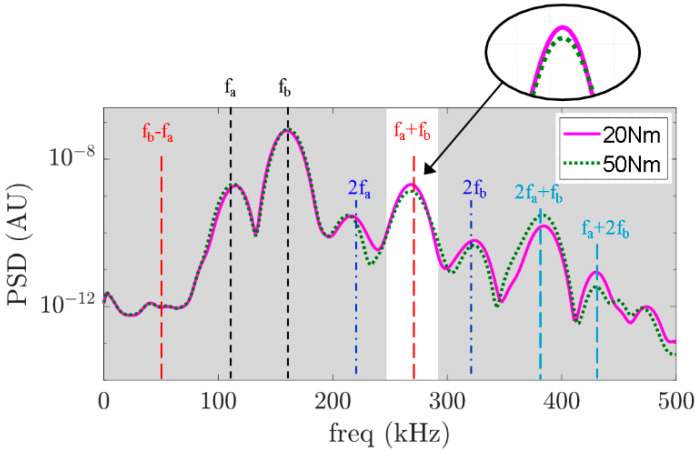
PSD comparison between two different applied torques.

However, time-domain features add extra complexity to the data analysis when complicated pulses and wave reflections are involved, especially when the incident wave is not a single-frequency pulse. It is hard to obtain useful information about the bolt condition from the time-domain signals directly. In this study, the data are analysed in a frequency domain. The power spectral density is calculated using the Welch periodogram for each measurement. Figure 7 shows the frequency domain of the signals shown in Figure 6. The signal contains frequency components at 110 kHz and 160 kHz. The presence of second harmonics at 220 kHz and 320 kHz and combinational harmonics at 50 kHz, 270 kHz, 380 kHz, and 430 kHz reveal the nonlinear features of the GWs in the frequency domain. By comparing the power spectrum, we can see that the power of the combinational harmonic at the sum frequency, which is 270 kHz, is weaker than that for the signal where the bolt is tightened. 

**Figure 8:**
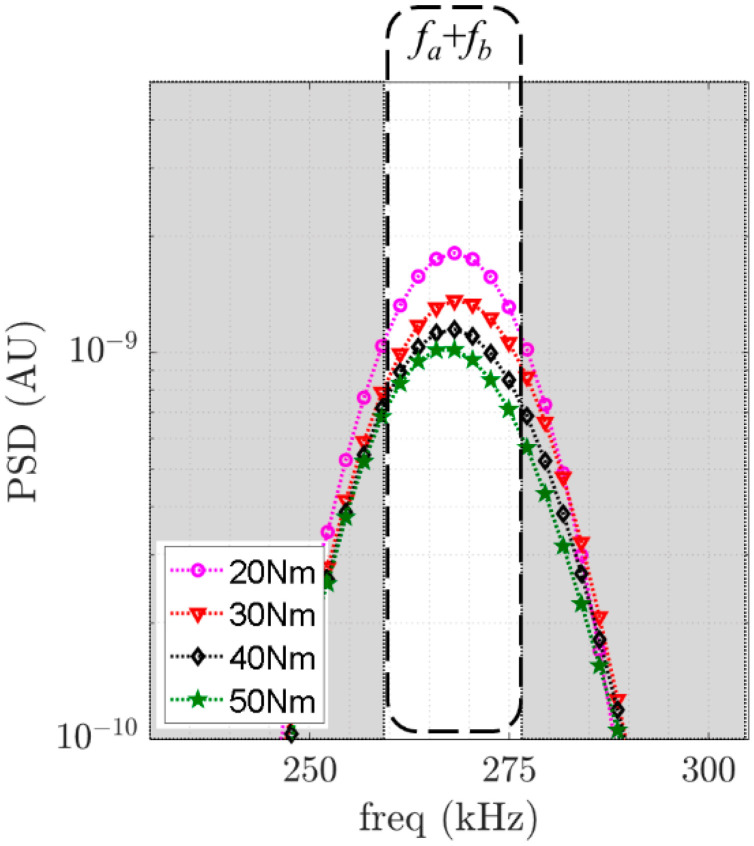
PSD comparison between four different levels of applied torque at the sum frequency component.

Using these two scenarios, we can intuitively predict that when the applied torque is increased from 20 Nm to 50 Nm, the contact interface between the washers and the plates also increases. For the case where the bolted joint is tighter at 50 Nm as compared to the case of 20 Nm, the contact effect produced as a consequence of the guided wave travelling through the bolted joint is lesser for the 50 Nm case; hence, the combinational harmonic for the 50 Nm torque is lower than that for the 20 Nm torque. Given the limitations on second harmonic generation already mentioned in the introduction section, such as the small magnitude of the second harmonic and equipment-related nonlinearities, this study focuses on combinational frequency component, specifically at the sum frequency. In this context, we calculate the power of each measurement for every torque value considered. In fact, it can be observed in Figure 8 that there is a relationship between the power of the sum frequency component and the applied torque. For clarity, only 4 of the 11 levels of torque are shown in Figure 8.

**Figure 9:**
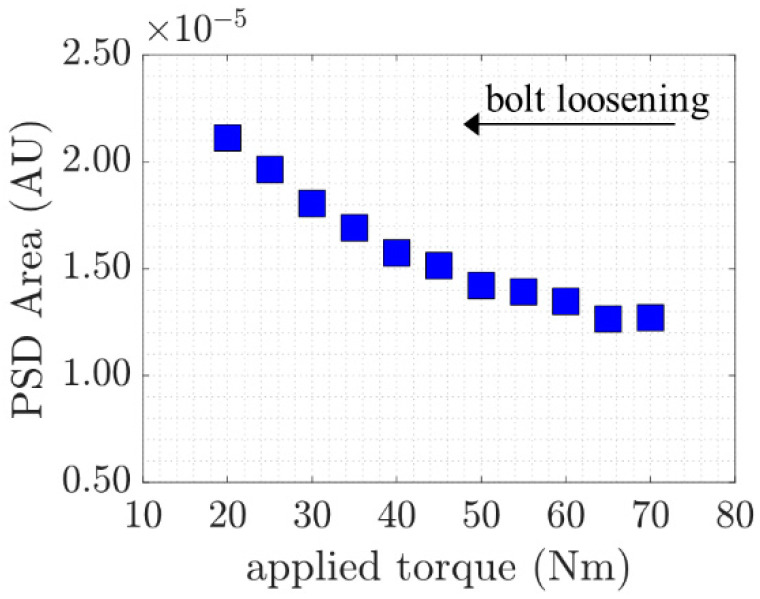
Combinational harmonic at sum frequency against applied torque.

To further demonstrate the proposed mixed-frequency technique in monitoring bolted joints, the relationship between the applied torque and the areas of power spectral density in the region of the combinational harmonic at the sum frequency within a 32 ± 4 kHz bandwidth were calculated for all five measurements. The averaged values are shown in Figure 9. A decreasing trend is notably observed. The results are consistent for all five measurements and show that the proposed approach can be used as an indicator for applied bolt torque.

As described before, when the bolt is tighter, the contact effect decreases. These steadily decreasing values provide a useful understanding of the nature of the combinational harmonic behaviour when a GW passes through a bolted joint, which provides valuable information for the condition of the applied torque. Moreover, the study also shows that the condition of the joint can be assessed by employing two inexpensive piezoelectric transducers without the need for complicated vibration or impact generator equipment.

## 5. Effect of the Number of Cycles

**Figure 10:**
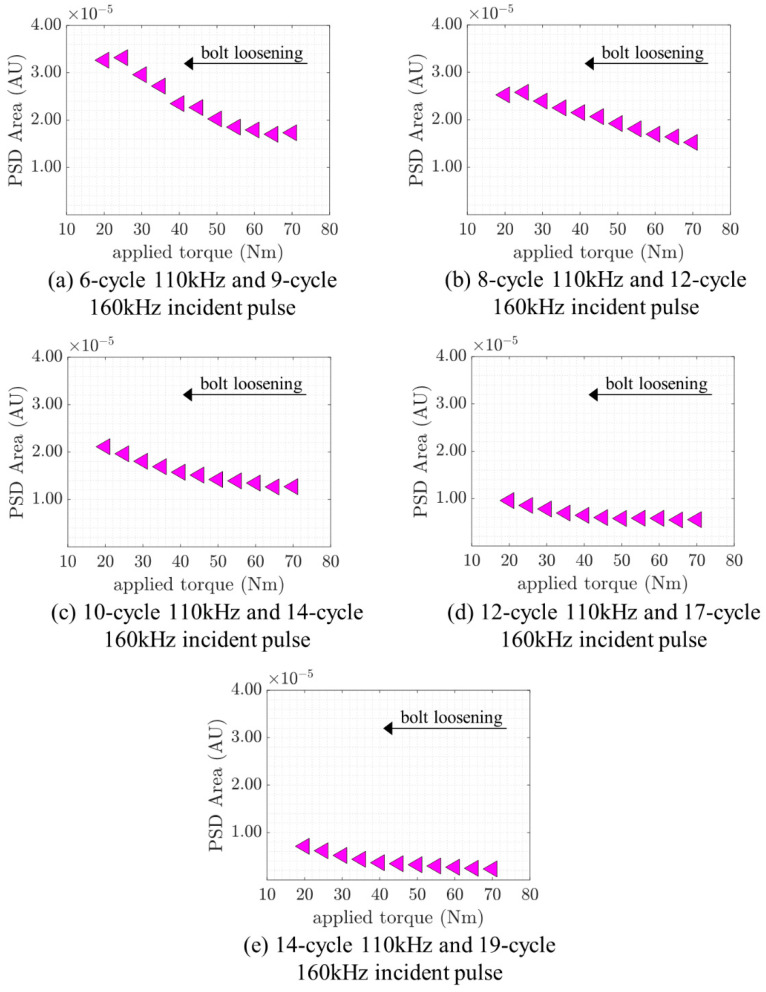
Relationship between PSD area and applied torque.

In this section, the effect of the duration/number of cycles of the incident wave is investigated. This study was conducted by increasing the number of cycles of the incident pulses. Apart from the signal studied previously (8-cycle 110 kHz signal with a 12-cycle 160 kHz signal), several different sinusoidal tone bursts were generated and measured by the actuator–sensor pair. The pairs consisted of a 6-cycle 110 kHz signal with a 9-cycle 160 kHz signal, an 8-cycle 110 kHz signal with a 12-cycle 160 kHz signal, a 12-cycle 110 kHz signal with a 17-cycle 160 kHz signal, and a 14-cycle 110 kHz signal with a 19-cycle 160 kHz signal. The numbers of cycles were selected so that both single-frequency sinusoidal tone-burst pulses for each pair would have the same duration and have similar energy content. The area under the curve in the power spectrum within a 32 ± 4 kHz bandwidth was calculated for all five frequency pairs. For each of the five frequency pairs, the calculated area values were plotted versus the applied torque in the same way as the previous section and are shown in Figure 10.

A decreasing trend is observed for all five frequency pairs. The proposed technique therefore shows that the applied torque in the bolted joint can be used to monitor the mixed-frequency signals regardless of the employed number of cycles. The trend shifts downwards as the number of cycles increases. The explanation for this phenomenon is that the bandwidth of the signal at the sum frequency component becomes sharper when the number of cycles is increased. In turn, when the frequency component reduces its bandwidth, the area under its curve decreases in magnitude, which shifts all values downwards. This provides a significant insight that even though the proposed monitoring technique is sensitive to the number of cycles, it does not affect its overall performance in monitoring the applied torque using the combinational frequency component.

**Figure 11:**
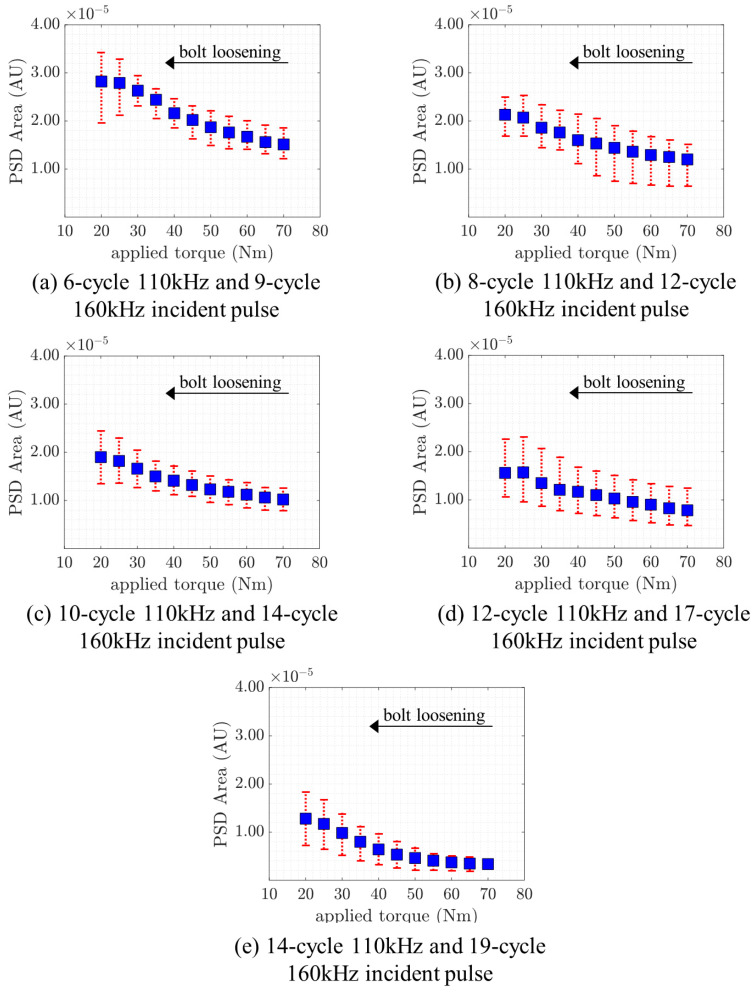
Mean, minimum, and maximum values of the PSD area against applied torque.

After processing the signal from all five measurements for each frequency pair, the average values with their respective maximum and minimum values were plotted, as seen in Figure 11. The steady decrease for all five cases further demonstrates the robustness of the proposed technique. Particularly, less uncertainty can be observed when the incident pulse is a 14-cycle 110 kHz and 19-cycle 160 kHz wave. In addition, a trend of decreasing variation is observed as applied torque increases. When the bolt becomes loose, many reflections occur as a result of a localized effect, whereas these reflections are less likely to occur and are lesser in magnitude as the torque increases. This phenomenon is echoed in the reduced variation with increasing applied torque.

## 6. Conclusions

With the increasing need to detect structural failures or underperformances in civil and mechanical engineering structures, this study has proposed a nonlinear GW-mixing approach to address the bolted-joint monitoring issue in steel plates. A theoretical development has been presented, according to which the applied torque is correlated to the combinational harmonic at sum frequency due to wave mixing. In this study, a signal containing two central frequency components has been used as input signal. The combinational harmonics at sum frequency are induced by contact acoustic nonlinearity of the bolted joints and have been studied under different levels of applied torque. The results show that the frequency spectra of the measured signals carry information on the bolted joint condition. This study has shown that the mixed-frequency signal is sensitive to the applied torque, and the combinational harmonic at sum frequency increases with a decreasing bolt torque, showing that the early bolt loosening can be detected. This study has also demonstrated the effect of the number of cycles of the incident signal on the combinational harmonic at sum frequency. The results show that the correlation between the applied torque and the combinational harmonic at sum frequency of the GW mixing approach is robust. In addition, by only employing small and inexpensive PZT transducers, this approach could pave the way as a future alternative for online bolted joints monitoring.

## Data Availability

Not applicable.
